# Effect of Olanzapine on Clinical and Polysomnography Profiles in Patients with Schizophrenia

**DOI:** 10.1155/2018/3968015

**Published:** 2018-02-20

**Authors:** Mohammad Zia Ul Haq Katshu, Sukanto Sarkar, S. Haque Nizamie

**Affiliations:** ^1^Institute of Mental Health, University of Nottingham, Nottingham, UK; ^2^Nottinghamshire Healthcare NHS Foundation Trust, Nottinghamshire, UK; ^3^Mahatma Gandhi Medical College and Research Institute, Pondicherry, India; ^4^K S Mani Centre for Cognitive Neurosciences, Central Institute of Psychiatry, Ranchi, India

## Abstract

Acute and short-term administration of olanzapine has a favorable effect on sleep in schizophrenia patients. This study aimed to clarify the effect of olanzapine on polysomnographic profiles of schizophrenia patients during the acute phase of illness after controlling for previous drug exposure. Twenty-five drug-naïve or drug-free schizophrenia patients were assessed at baseline and after six weeks of olanzapine treatment on Brief Psychiatric Rating Scale (BPRS), Positive and Negative Syndrome Scale (PANSS), and Udvalg for Kliniske Undersogelser (UKU) side-effect rating scale and a whole-night polysomnography; fifteen patients completed the study. There was a significant reduction in all psychopathological variables with maximum reduction in PANSS total, BPRS total, and PANSS positive scores. A significant increase in total sleep time (TST), sleep efficiency (SE), nonrapid eye movement (NREM) stage 1 duration, stage 3 duration, stage 4 duration, and stage 4 percentage of TST, number of rapid eye movement (REM) periods, REM duration, and REM percentage of TST was observed. REM latency at baseline inversely predicted the reduction in BPRS total and PANSS total and positive scores. In summary, short-term treatment with olanzapine produced significant improvement in clinical and polysomnography profiles of patients with schizophrenia with shorter REM latency predicting a good clinical response.

## 1. Introduction

Sleep remains disturbed in around 30–80% patients with schizophrenia [1]. The degree of impairment in sleep may be a reflection of the severity of symptoms. Prolonged periods of total sleeplessness may be seen during psychotic agitation. Less severe symptoms may result in longer sleep onset latency, reduced total sleep time (TST), and sleep fragmented by bouts of awakening [2, 3]. Besides the overall quantitative changes, the architecture of sleep also changes significantly. A significant decrement in rapid eye movement (REM) latency, REM duration, REM percentage of TST, nonrapid eye movement (NREM) stage 2, and slow wave sleep (SWS), in addition to a reduction in delta power, has been observed in most studies [2–4].

Antipsychotics, the mainstay of pharmacotherapeutic treatment in schizophrenia, exert variable effects on sleep, depending on their receptor profiles. Typical antipsychotics like haloperidol, thiothixene, and flupentixol increase TST and Sleep Efficiency (SE) and reduce Sleep Latency (SL) and awakenings [5, 6]. Atypical antipsychotics have a more favorable impact on sleep profiles. Clozapine [7] improves SE and increases stage 2 NREM sleep, while risperidone [6] increases SWS also. Of the atypical antipsychotics, olanzapine holds the promise of the most favorable effects on sleep. Single dose (5 mg/10 mg) administration of olanzapine in healthy individuals increased SE and SWS [8, 9], more so in females [10], and also showed an increase in REM sleep [11]. Olanzapine showed similar increase in SE and SWS after single dose administration in depressed patients which was maintained over 3 weeks of treatment [12]. The effect of olanzapine on sleep in schizophrenia patients has been the subject of very few studies. Salin-Pascual et al. [13, 14] found an increase in TST, stage 2 NREM, and SWS and a reduction in awakenings and stage 1 NREM sleep after acute administration of olanzapine (10 mg) for two consecutive nights. Müller et al. [15] reported an increase in SE, SWS, and REM sleep after four-week administration of olanzapine (15–20 mg) in ten schizophrenia patients. Considering the observations by Tandon et al. [16] that measurements of polysomnography change over first four weeks of medication withdrawal and tend to stabilize after more than four weeks, the four-day minimum drug free period seems inadequate to control for the effects of medication in the study by Müller et al. [15]. Also, the study population consisted of residual and disorganized schizophrenia subtypes making it difficult to generalize these findings. Göder et al. [17] found an increase in SWS, but no change in memory, after a single dose of olanzapine in patients with schizophrenia. More recently, Kluge et al. [18] showed that olanzapine resulted in a significantly higher increase in SWS compared to clozapine.

The effect of olanzapine on sleep profiles in schizophrenia patients is important from a clinical perspective as improvements in the overall quantity and quality of sleep are related to sociooccupational functioning of patients. Furthermore, changes in sleep architecture with olanzapine may provide a better understanding of its pharmacodynamic profile as well as help understand the neurobiology of schizophrenia. Considering this, we carried out a six-week prospective study on the effect of olanzapine on polysomnography profiles of schizophrenia patients in an acute phase of illness. Also, changes in psychopathological scores with six weeks of olanzapine treatment and correlations between changes in psychopathological scores and sleep profiles were examined. This study was part of a larger project, part of which has been reported earlier [4].

## 2. Materials and Methods

### 2.1. Participants

Our study population consisted of twenty-five, right-handed male patients who were drug naïve/free (for at least four months) at the time of study. All the patients were in the age group of 18–45 years meeting the ICD-10 DCR [19] diagnostic criteria for schizophrenia. Patients having comorbid psychiatric, neurological, or medical illness and substance use (excluding nicotine and caffeine) were excluded from the study. Patients were recruited through purposive sampling from the inpatient services of the Central Institute of Psychiatry (Ranchi, India), a tertiary treatment and research centre. All the participants provided written informed consent to take part in the study. The study was approved by the institute's ethics review committee.

### 2.2. Measures

Sociodemographic and clinical data sheet was used to collect the relevant information. The Sleep Disorders Questionnaire (SDQ) [20], Hindi version of Sidedness Bias Schedule (SBS) [21], and Calgary Depression Rating Scale (CDSS) [22] were used to screen out presence of any primary sleep disorder, left-handedness, and depression in all the patients, respectively. For assessment of psychopathology, Brief Psychiatric Rating Scale (BPRS) [23] and Positive and Negative Syndrome Scale (PANSS) [24] were used. The side effects were assessed using the Udvalg for Kliniske Undersogelser (UKU) [25].

### 2.3. Procedure

Patients were briefed about the research study and its purpose and their consent to participate in the study was taken. Patients were selected only after they were screened for handedness using Hindi version of SBS, primary sleep disorders by SDQ, and depression using the CDSS, respectively. BPRS, PANSS, and UKU ratings and a whole-night polysomnography were recorded from patients at baseline and after six weeks of olanzapine treatment. Five patients could not complete the baseline assessment, specifically the whole-night polysomnography, and five more patients did not turn up for the second assessment and thus had to be dropped out from the study.

### 2.4. Polysomnography Recording

Sleep study was done in the sleep laboratory of the Centre for Cognitive Neurosciences, Central Institute of Psychiatry (Ranchi, India). The procedure of whole-night polysomnography has been described previously [4]. Staging of sleep was done manually using central (C3, C4) and occipital (O1, O2) electrode placements referenced to linked ears (A2, A1), in 30-second epochs, following the Rechtschaffen and Kales (RK) criteria [26]. RK criteria were used to allow comparison with previous studies. Before scoring for sleep stages, polysomnographs were screened for the presence of sleep apnea and periodic movements in sleep as defined by International Classification of Sleep Disorders (2nd ed.) [27]. None of the fifteen patients who completed the study met criteria for either sleep apnea or periodic movements in sleep.

### 2.5. Statistical Analysis

Statistical Package for Social Sciences version 10.0 was used for statistical analysis (SPSS, Inc., Chicago, Illinois). The categorical and continuous variables characterizing the sample were described using descriptive statistics. Paired-samples *t*-test was used to compare the pre- and post-treatment psychopathological scores. As the polysomnographic data were not normally distributed (Shapiro-Wilk test), Wilcoxon Signed-Ranks test (Monte Carlo method) was used to compare the pre- and post-treatment polysomnography measures. The effect size, Cohen *d*, was computed using standard deviations (SDs) of pre- and post-treatment measures as suggested by Dunlop et al. [28] for correlated designs. Spearman's correlation coefficients (*r*_*s*_) were calculated between the baseline polysomnography parameters and psychopathology scores. Linear regression (forced entry method) was used to assess the relationship between baseline polysomnography parameters and the reduction in psychopathological scores.

## 3. Results

### 3.1. Sociodemographic and Clinical Measures

Table 1 summarises the sample characteristics. The mean age of onset of illness was 23.8 (SD 4.5) years and the mean illness duration was 34.6 (SD 33.9) months. Around two-thirds (65%) of patients had paranoid schizophrenia and the rest undifferentiated schizophrenia. Of the twenty patients who completed the baseline assessment, eleven were drug naïve while nine were drug free with a minimum drug free interval of seven months. All the patients received olanzapine in a flexible dosage regime with a mean dosage of 19 milligrams (SD 3.87). The most common side effects experienced were sedation (60%), rigidity (20%), and tremor (20%).

### 3.2. Effect of Olanzapine on Psychopathological Scores

Comparison of pre- and posttreatment psychopathological scores is given in Table 2. There was a significant reduction in all the psychopathological variables with maximum reduction in PANSS total (*p* < 0.001, *d* = 4.35), BPRS total (*p* < 0.001, *d* = 3.84), and PANSS positive (*p* < 0.001, *d* = 3.13) followed by PANSS general (*p* < 0.001, *d* = 3.03) and least but significant improvement in PANSS negative scores (*p* < 0.001, *d* = 0.79).

### 3.3. Effect of Olanzapine on Polysomnography Parameters

Comparison of the pre- and post-treatment polysomnography parameters is given in Table 3. Treatment with olanzapine over the 6-week period resulted in significant increase in TST (*p* = 0.005, *d* = −1.43), SE (*p* = 0.004, *d* = −1.0), NREM stage 1 duration (*p* = 0.029, *d* = −1.1), NREM stage 3 duration (*p* = 0.025, *d* = −0.91), NREM stage 4 duration (*p* = 0.005, *d* = −0.83), NREM stage 4 percentage of TST (*p* = 0.034, *d* = −0.66), number of REM periods (*p* = 0.004, *d* = −1.25), REM duration (*p* = 0.001, *d* = −1.27), and REM percentage of TST (*p* = 0.004, *d* = −1.1).

### 3.4. Correlation between Polysomnography and Psychopathological Scores

**Figure 1:**
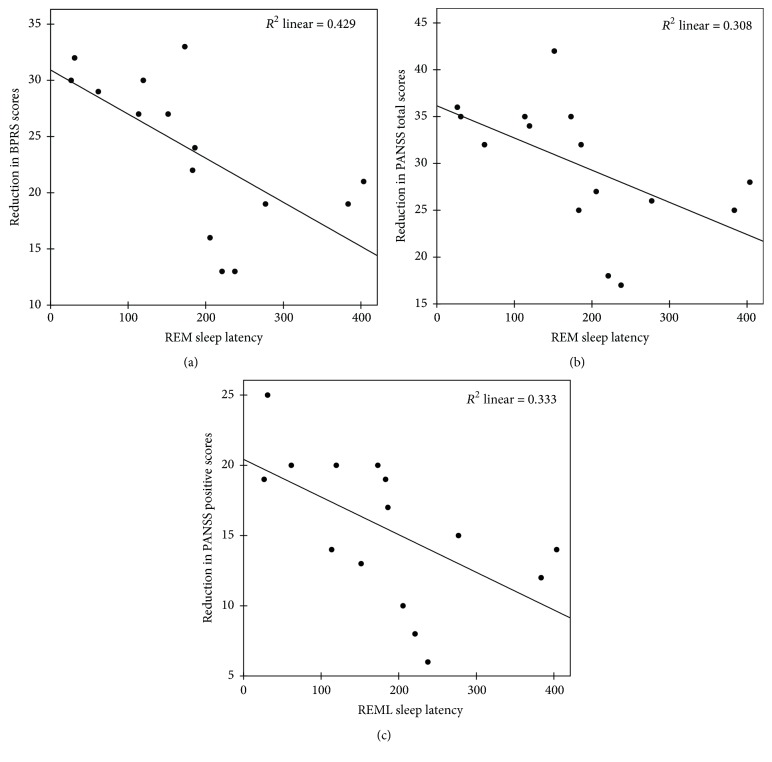
Scatter plot showing significant negative correlations between REM latency at baseline and reduction in BPRS total scores (a), reduction in PANSS total scores (b), and reduction in PANSS positive scores (c).

Correlations between baseline sleep parameters and psychopathological scores have been reported previously in [4]. In summary, REM percentage of TST showed a significant positive correlation with BPRS total score (*r*_*s*_ = 0.488, *p* = 0.029) and PANSS positive score (*r*_*s*_ = 0.583, *p* = 0.007). Also, REM latency showed a significant negative correlation with BPRS total score (*r*_*s*_ = −0.640, *p* = 0.002) and PANSS positive score (*r*_*s*_ = −0.657, *p* = 0.002). Based on findings from previous studies [4, 14, 29, 30] and after excluding sleep parameters with high correlations, REM latency and NREM stage 4 duration were entered as independent variables to predict reduction in psychopathological scores in linear regression analysis. REM latency inversely predicted the reduction in BPRS total scores from baseline (*β* = −0.655, *p* = 0.008) explaining 42.9% of variance, the reduction in PANSS total scores from baseline (*β* = −0.555, *p* = 0.032) explaining 30.8% of variance, and the reduction in PANSS positive scores (*β* = −0.577, *p* = 0.024) explaining 33.3% of variance (Figure 1).

## 4. Discussion

Our study evaluated the effect of short-term treatment of olanzapine in schizophrenia patients with predominantly positive symptoms. The study controlled the previous medication status, by recruiting drug naïve or drug free patients with a minimum drug free interval of 4 months (actual minimum drug free period was 7 months), which could have affected the results of previous studies [15, 18]. Our study screened out depression by using CDSS, as sleep profiles of depressive patients are quite similar to schizophrenia patients [31] and recently olanzapine has been shown to increase SE and SWS in SSRI resistant depression [12]. The effect of olanzapine was studied over six-week duration to provide adequate time for clinical improvement and stabilization of any changes in sleep profiles.

Of the 15 patients that completed the study, 13 were responders, taking 30% reduction in baseline BPRS scores as a response criterion [13]. There was a differential improvement in different psychopathological sores with maximum improvement in positive symptoms (15 responders with a mean reduction of 16.46 points on PANSS positive subscale) followed by general psychopathological symptoms (7 responders with a mean reduction of 10.54 points on PANSS general subscale), with the least but significant improvement in negative symptoms (7 responders with a mean reduction of 3.8 points on PANSS negative subscale). These findings are quite consistent with the earlier studies of olanzapine showing a greater improvement in positive symptoms and little but significant improvement in negative symptoms [32]. The most common side-effect observed was sedation (60%) followed by extrapyramidal symptoms (EPS, 20%), with constipation and orthostatic hypotension being the least (6.7% each). The relatively higher rates of EPS observed may be attributed to higher dosages of olanzapine needed (dose range of 15–25 milligrams, mean 19 milligrams) in patients which is consistent with earlier studies showing a dose-dependent increase in EPS. Metabolic side effects including weight gain, insulin resistance, and changes in lipid profile were not assessed as part of the study.

The study showed improvement in sleep maintenance with an increase in SE, TST, SWS, and REM sleep consistent with previous studies [15, 18]. An increase in SE and SWS has also been found after single dose of olanzapine administration in healthy individuals [8–11], in SSRI resistant depressed patients who were treated for over three weeks with olanzapine [12], and in schizophrenia patients after acute administration of olanzapine [13, 14, 17]. A decrease in REM duration and number of REM periods has been reported by Sharpley et al. [8], while an increase in REM sleep was reported by Giménez et al. [11] after a single dose of olanzapine in healthy volunteers. The changes in sleep measures following olanzapine administration may reflect either a reduction of acute psychosis, a direct medication effect unrelated to the antipsychotic effect, or both. As the studies involving a single dose administration of olanzapine have shown similar changes in the sleep measures not only in schizophrenia patients but in depressed patients and healthy individuals, the changes seem to be better explained by direct medication effects unrelated to the antipsychotic effect.

The improvement in sleep continuity measures may be explained by H1 antagonistic properties of olanzapine, similar to the effects observed with H1 antagonistic agents like mepyramine [33, 34]. In addition, it is possible that 5-HT2A/2C receptor blockade could contribute to improvement in sleep continuity, as similar effects have been observed with 5-HT2 receptor antagonist nefazodone [34, 35]. The improvement in NREM sleep, mainly in SWS by olanzapine, has been attributed to 5HT2 antagonism. 5-HT2 receptor antagonists like risperidone, ritanserin, selective 5-HT2A receptor antagonist (M100907), and selective 5-HT2C antagonist 6-chloro-5-methyl-1-(5-quinolylcarbamoyl) indoline (SB-243213) greatly enhance SWS [6, 36–38], while 5-HT2 agonists, 1-(2,5-dimethoxy-4-iodophenyl)-2-aminopropane (DOI) and 1-(3-chlorophenyl) piperazine (m-CPP), and selective 5HT2B receptor antagonist (SB-215505) increase wakefulness and decrease slow wave sleep [39, 40]. These data indicate that SWS enhancing effect of olanzapine may be mediated by 5-HT2A and/or 5-HT2C receptors. Studies on the effect of olanzapine on REM sleep are inconsistent. While antagonistic effects of olanzapine on muscarinic cholinergic receptors would predict a decrease in the REM sleep, 5HT2 agonists, 1-(2, 5-dimethoxy-4-iodophenyl)-2-aminopropane (DOI) and 1-(3-chlorophenyl) piperazine (m-CPP), decrease REM sleep, and cyproheptadine, 5-HT2A receptor antagonist, increases REM sleep [39–42]. Decrease in serotonergic inhibition during NREM sleep of cholinergic cell groups giving rise to Ponto-geniculo-occipital (PGO) waves may provide a possible mechanism for the increase in REM sleep with olanzapine through 5HT2 antagonism [43, 44].

The deficits in slow wave sleep are said to represent a trait-like abnormality [4, 45]. One of the arguments put forth for this is the persistent nature of slow wave sleep deficits, unaffected by treatment. The results of our study and previous studies [12, 15, 18] have shown significant increases in slow wave sleep after treatment with olanzapine, thereby, questioning this line of thinking. Long term follow-up studies are needed to clarify whether there are both state and trait components to the SWS deficits observed in schizophrenia. On the other hand these findings point to the need for redefining trait-like abnormalities, especially with regard to their nonresponse to treatment nature.

Shorter REM latency predicted a good response to olanzapine at six weeks of treatment in our study, which seems apparently inconsistent with the only comparable previous study showing SWS deficit associated with a better short-term response to olanzapine [14]. Although shorter REM latency in schizophrenia patients is a consistent observation, opinions differ as to the underlying mechanism. Short REM latency could represent an intrusion of REM sleep into NREM sleep [46], early onset of the first REM period [47], or a SWS deficit in the first NREM period that permits the passive advance. Research evidence does not support the REM intrusion hypothesis [46], while SWS deficit has been found consistently in schizophrenia patients [2, 3] and seems to be the likely explanation for shorter REM latency which perhaps explains the apparently contradictory findings of the present study and the previous study by Salin-Pascual et al. [14]. Also, REM latency shows a negative correlation with severity of psychopathology and positive symptoms [4, 16], which are predictors of good response to antipsychotic treatment [48], supporting the results of our study. Further, SWS deficit has been associated with increased ventricular volume and ventricle-to-brain ratio [49] which predicts better response to olanzapine directly [50] as well as through its association with more severe positive symptoms [51]. However, SWS deficit has also been associated with more negative symptoms which predict a poorer treatment response [16, 52]. Again, more severe psychopathology and positive symptoms have also been found to predict a poor outcome [48]. The results also seem inconsistent with studies showing SWS deficit and shorter REM latency as predictors of poor long term outcome [29, 30]. These studies used different outcome measures, did not evaluate response to any specific antipsychotic medication, and had more heterogeneous samples that limit comparison with the present study.

One of the major limitations of our study was that analysis was done on first night polysomnography data, which may also explain the low mean TSP and higher percentage of Stage 1 sleep. However, the changes in polysomnography profiles following treatment observed in our study are similar to those reported from studies carried out on second or third night [11, 12, 15, 17, 18]. Also, both pre- and post-treatment recordings were of first night only and that may have served as an effective control per se. Furthermore, first night effect has been reported to affect schizophrenia patients significantly less (only 35%) than healthy controls (80%) [53]. Nevertheless, as Jobert et al. [54] have reviewed, first night effect remains an important limitation of the study. Another limitation of the study was lack of a control group. Further studies with adaptation nights, larger sample sizes, and control groups need to confirm these findings.

## Figures and Tables

**Table 1:** Sociodemographic and clinical characteristics of the patients (*N* = 15).

Sociodemographic/clinical variable	
Age (years), mean (SD)	26.33 (6.08)
Duration of illness (months), mean (SD)	32.80 (29.85)
Age of onset (years), mean (SD)	23.67 (4.92)
Drug free period (*N* = 7) (months), mean (SD)	31.85 (35.50)
Olanzapine dosage (milligrams), mean (SD)	19.0 (3.87)
Education, *n* (%)	
<10 years	7 (46.6)
>10 years	8 (53.4)
Occupation, *n* (%)	
Employed	11 (83.3)
Unemployed	4 (26.7)
Marital status, *n* (%)	
Single	10 (46.7)
Married	5 (33.3)
Family income, *n* (%)	
Low	14 (93.3)
High	1 (6.7)
Family type, *n* (%)	
Nuclear	4 (26.7)
Joint	11 (73.3)
Residence, *n* (%)	
Rural	12 (80.0)
Urban	3 (20.0)
Family psychiatric illness, *n* (%)	
Non-affective	5 (33.3)
Affective	1 (6.7)
Schizophrenia subtype, *n* (%)	
Paranoid	9 (60.0)
Undifferentiated	6 (40.0)
Drug status, *n* (%)	
Drug naive	8 (53.3)
Drug free	7 (46.7)
Side-effects, *n* (%)	
Sedation	9 (60)
Rigidity	3 (20)
Tremor	3 (20)
Constipation	1 (6.7)
Orthostatic hypotension	1 (6.7)

**Table 2:** Comparison between pre- and posttreatment psychopathological scores (*N* = 15).

Psychopathological variables	Pretreatment (Mean ± SD)	Posttreatment (Mean ± SD)	*T* (dF = 14)	*P* (2-tailed)
BPRS total	56.80 ± 8.14	33.13 ± 3.09	13.69	**<0.001** ^**∗∗**^
PANSS total	75.53 ± 8.38	45.73 ± 4.85	16.68	**<0.001** ^**∗∗**^
PANSS positive	25.13 ± 6.65	9.67 ± 2.13	11.53	**<0.001** ^**∗∗**^
PANSS negative	16.13 ± 5.83	12.33 ± 3.39	4.93	**<0.001** ^**∗∗**^
PANSS general	34.27 ± 4.30	23.73 ± 2.37	10.16	**<0.001** ^**∗∗**^

^*∗∗*^ *p* < 0.01.

**Table 3:** Comparison of pre- and posttreatment polysomnography parameters (Wilcoxon Signed-Ranks Test–Monte Carlo Method; *N* = 15).

Sleep variables	Pretreatment (Mean ± SD)	Posttreatment (Mean ± SD)	*Z*	*p* (2-tailed)
Total sleep period (min)	380.43 ± 113.80	440.90 ± 41.55	−1.278	0.210
Total sleep time (min)	263.83 ± 128.83	391.32 ± 47.15	−2.669	0.005^**∗∗**^
Sleep efficiency (%)	64.01 ± 23.36	82.38 ± 10.27	−2.784	0.004^**∗∗**^
Sleep onset latency (min)	12.90 ± 20.32	21.68 ± 12.90	−1.420	0.169
Stage 1 shifts	22.33 ± 9.22	26.00 ± 5.70	−0.712	0.486
Stage shifts	78.40 ± 32.40	84.53 ± 25.90	−0.142	0.896
Awakenings	13.93 ± 8.78	10.13 ± 8.32	−1.279	0.214
Stage 1 duration	70.10 ± 44.18	106.96 ± 28.02	−2.158	0.029^**∗**^
Stage 1 total sleep time (%)	29.90 ± 16.82	27.56 ± 7.08	−0.114	0.931
Stage 2 duration	124.40 ± 88.61	140.20 ± 63.28	−0.454	0.672
Stage 2 total sleep time (%)	45.94 ± 18.61	35.31 ± 14.70	−1.676	0.099
Stage 2 latency	25.93 ± 67.12	17.66 ± 27.02	−1.307	0.204
Stage 3 duration	30.63 ± 20.88	50.60 ± 27.0	−2.215	0.025^**∗**^
Stage 3 total sleep time (%)	11.56 ± 8.25	13.52 ± 8.81	−0.511	0.634
Stage 3 latency	96.40 ± 84.70	49.63 ± 50.58	−1.350	0.195
Stage 4 duration	3.23 ± 5.54	11.02 ± 11.92	−2.668	0.005^**∗∗**^
Stage 4 total sleep time (%)	1.16 ± 2.20	2.83 ± 3.0	−2.080	0.034^**∗**^
Stage 4 latency	166.92 ± 125.10	72.62 ± 52.73	−1.992	0.059
Number of REMs	2.47 ± 1.35	3.93 ± 1.22	−2.790	0.004^**∗∗**^
REM duration	35.47 ± 36.30	82.53 ± 45.94	−3.067	0.001^**∗∗**^
REM total sleep time (%)	11.43 ± 8.65	20.77 ± 10.32	−2.755	0.004^**∗∗**^
REM latency	169.82 ± 118.11	120.66 ± 49.64	−1.287	0.214

^*∗*^ *p* < 0.05, ^*∗∗*^*p* < 0.01.
